# MAPK4 silencing in gastric cancer drives liver metastasis by positive feedback between cancer cells and macrophages

**DOI:** 10.1038/s12276-023-00946-w

**Published:** 2023-02-17

**Authors:** Shuang Li, Dongyang Guo, Qiang Sun, Lu Zhang, Yun Cui, Min Liu, Xixi Ma, Yiman Liu, Wenyu Cui, Leimin Sun, Lisong Teng, Liangjing Wang, Aifu Lin, Wei Liu, Wei Zhuo, Tianhua Zhou

**Affiliations:** 1 Department of Cell Biology and Cancer Institute of the Second Affiliated Hospital, Zhejiang University School of Medicine, Hangzhou, China; 2 Cancer Center, Zhejiang University, Hangzhou, China; 3 Institute of Gastroenterology, Zhejiang University School of Medicine, Hangzhou, China; 4 Department of Oncology, First Affiliated Hospital of Zhejiang University School of Medicine, Hangzhou, China; 5 Department of Gastroenterology, Second Affiliated Hospital of Zhejiang University School of Medicine, Hangzhou, China; 6 College of Life Sciences, Zhejiang University, Hangzhou, China; 7 Department of Biochemistry, and Department of Cardiology of the Second Affiliated Hospital, Zhejiang University School of Medicine, Hangzhou, China; 8 Department of Molecular Genetics, University of Toronto, Toronto, ON Canada

**Keywords:** Metastasis, Gastric cancer, Cancer microenvironment

## Abstract

Liver metastasis is a major cause of death in gastric cancer patients, but the underlying mechanisms are poorly understood. Through a combination of in vivo screening and transcriptome profiling followed by quantitative RT-PCR and tissue array analyses, we found that mitogen-activated protein kinase 4 (MAPK4) downregulation in gastric cancer tissues from patients is significantly associated with liver metastasis and poor prognosis. The knockdown of MAPK4 in gastric cancer cells promotes liver metastasis in orthotopic mouse models. MAPK4 depletion in gastric cancer cells induces the secretion of macrophage migration inhibitory factor (MIF) to polarize tumor-associated macrophages (TAMs) in orthotopic xenograft tumors. Moreover, TAMs activate epithelial–mesenchymal transition of gastric cancer cells to suppress MAPK4 expression, which further increases MIF secretion to polarize TAMs. Taken together, our results suggest a previously undescribed positive feedback loop between cancer cells and macrophages mediated by MAPK4 silencing that facilitates gastric cancer liver metastasis.

## Gastric cancer: Gene shutdown helps spread to the liver

Reduced activity of a key cell control gene mediates an interaction between gastric cancer cells and immune system cells called macrophages that allows the cancer to spread to the liver. Cancer cells migrating to the liver is a major cause of death for gastric cancer patients, but the mechanisms driving this process have been unclear. Researchers in China led by Tianhua Zhou and Wei Zhuo at Zhejiang University School of Medicine, Hangzhou, studied the activity of genes and their corresponding proteins in patients’ cancer cells, and analyzed gene activity in mouse models of gastric cancer. They found that the gene that generates a protein called MAPK4 is significantly reduced in the cancer cells. This allows the cells to interact with macrophages, promoting their spread to the liver.

## Introduction

Gastric cancer is the fifth most frequently diagnosed cancer and the fourth leading cause of cancer-related deaths worldwide^1^. Distant metastasis accounts for most gastric cancer-related deaths, and the 5-year survival rate of metastatic gastric cancer patients is below 5%^2–4^. The liver is the most common site for distant metastasis of gastric cancer^3,4^. However, the mechanisms underlying gastric cancer liver metastasis remain largely unknown.

Emerging evidence indicates that cancer metastasis is significantly facilitated by the interaction between cancer cells and stroma^5,6^. Tumor-associated macrophages (TAMs) have been recognized as a critical component of the tumor microenvironment^7,8^. Clinical studies show that an increased density of TAMs is strongly associated with a higher risk of cancer metastasis and poor prognosis in many cancer types, including gastric cancer^9–11^. Many studies suggest that communication between cancer cells and TAMs promotes metastasis in breast, pancreas, and lung cancers^12–15^. However, little is known about the role of the interaction between cancer cells and TAMs in gastric cancer metastasis.

Here, we employed an in vivo selection system to identify mitogen-activated protein kinase 4 (MAPK4) as a previously uncharacterized suppressor of gastric cancer liver metastasis in orthotopic mouse models. MAPK4 downregulation in gastric cancer tissues from patients was found to be significantly associated with liver metastasis and poor prognosis. The depletion of MAPK4 in gastric cancer cells induced the secretion of macrophage migration inhibitory factor (MIF) to polarize TAMs in orthotopic xenograft tumors. TAM polarization further suppressed MAPK4 expression in gastric cancer cells, suggesting a hitherto undescribed positive feedback loop between cancer cells and macrophages in gastric cancer liver metastasis.

## Materials and methods

### Clinical samples

Eighty-five paired frozen primary gastric tumor tissues and adjacent nontumor tissues were obtained from the Second Affiliated Hospital, Zhejiang University School of Medicine (Hangzhou, China). Tissue microarrays composed of 83 paired primary gastric tumor tissues and adjacent nontumor tissues were also acquired from the Second Affiliated Hospital, Zhejiang University School of Medicine (Hangzhou, China). Six paired frozen liver metastatic tissues and matched primary tumor tissues were obtained from the First Affiliated Hospital, Zhejiang University School of Medicine (Hangzhou, China). The clinical samples were collected with informed consent from the patients, and the experiments involving patient samples were approved by the Ethics Committee of Zhejiang University.

### Animal models

Female 4- to 6-week-old severe combined immunodeficiency (SCID) mice were purchased from the Vital River Laboratory (Beijing, China). For the tumor growth assay, 5 × 10^6^ cells were injected subcutaneously into the mice as described previously^16^. The tumor volumes were measured with a caliper and calculated using the following equation: *V* = (*L* × *W* × *W*)/2, where *V* is the tumor volume, *L* is the tumor length and *W* is the tumor width. For the orthotopic implantation experiment, the subcutaneous tumors were cut into pieces with a diameter of approximately 2 mm, and the pieces were then implanted on the stomach wall of the mice to generate liver metastases. The development of orthotopic tumors was assessed by palpation. The number of liver metastatic nodules was counted by visual observation after mouse dissection, and the number of liver micrometastases was calculated from liver sections with hematoxylin and eosin staining under microscopy. All experiments involving mice were approved by the Animal Care and Use Committee of Zhejiang University.

### Cell lines

BGC-823 cells were obtained from the Institute of Biochemistry and Cell Biology, Chinese Academy of Sciences (Shanghai, China)^17^, and MKN45 cells were acquired from the Chinese Academy of Medical Sciences (Beijing, China)^18^. PDX122 cells were established from a gastric cancer patient-derived xenograft model (kind gift from Dr. Lisong Teng, Zhejiang University School of Medicine, Hangzhou, China). All cancer cells were maintained in 1640 medium (Corning) supplemented with 10% fetal bovine serum (FBS) (HyClone) and penicillin/streptomycin. All the cells were confirmed to be negative for mycoplasma contamination. To block the proteolytic activity of the proteasome complex, cancer cells were incubated with 10 μM MG132 (Sigma) for 6 h. To inhibit protein synthesis, cancer cells were treated with 50 µg/ml cycloheximide (Sigma) for the indicated times.

### In vivo selection

To generate a highly metastatic variant from BGC-823 cells, we performed an in vivo selection experiment as described previously with some modifications^19^. Briefly, we implanted a subcutaneous tumor piece of BGC-823 cells on the stomach wall of the SCID mice to establish the orthotopic xenograft model. After 4–5 weeks, liver metastatic cells were isolated, expanded in culture, and then reinoculated into SCID mice for a second round of in vivo selection. BGC-LM cells were obtained from visible liver metastasis after three rounds of in vivo selection. Mouse fibroblasts were removed by differential trypsinization three times as described previously^20^.

### RNA sequencing

Total RNA was extracted using the TRIzol reagent (Invitrogen). Sequencing libraries were generated using the rRNA-depleted samples by the NEBNext Ultra Directional RNA Library Prep Kit for Illumina (NEB) following the manufacturer’s recommendations. The library quality was measured on an Agilent 2100 Bioanalyzer with respect to the product size and concentration. Paired-end libraries were sequenced using the Illumina HiSeq 2500 platform (read length of 150 bp). Fastp (v 2.2.0) was applied to remove adapters and low-quality reads. Paired-end reads were aligned to the human genome (hg19) with Hisat2 (v 2.1.0). StringTie (v 1.3.4) was used to calculate the FPKMs of each gene in each sample. The differential expression of genes was determined using DEseq2. Genes with *P* value < 0.05 and |log2 (fold change)| > 1 were considered significantly differentially expressed. The RNA sequencing data generated in our study are available in the Sequence Read Archive (SRA) database (accession number: PRJNA578582).

The gene profiling data of gastric cancer liver metastases and corresponding primary tumors were downloaded from the GEO database under the accession number GSE11072. We filtered out the probes mapped to multiple genes and calculated the average fold change for multiple probes targeting the same gene. *P* values < 0.05 and |log2 (fold change)| > 1 were considered to indicate genes that were significantly associated with liver metastasis (Student’s *t* test).

### Cell invasion and coculture system of cancer cells and BMDMs

For the cell invasion assay, cancer cells were seeded in the upper chamber precoated with Matrigel (Corning) in Boyden chambers. The lower chamber was filled with 10% FBS medium with or without BMDMs. The invaded cancer cells were stained with crystal violet (Sigma) and quantified.

To obtain cell samples from the coculture model for qRT-PCR or western blotting, cancer cells and BMDMs were seeded in the upper and lower chambers of Boyden chambers, respectively^21^. The cancer cells and BMDMs were harvested after 24 h and subjected to qRT-PCR and western analyses.

### Flow cytometry

A single cancer cell solution was prepared in PBS and incubated with an anti-mouse CD16/CD32 antibody (BD Biosciences) to block Fc receptors. The cells were washed with staining buffer and incubated with fluorescent-conjugated anti-mouse F4/80 (1:100, 123116, BioLegend) and fluorescent-conjugated anti-mouse CD206 (1:100, 141706, BioLegend). The cells were then diluted to an appropriate concentration and subjected to flow cytometry with an LSRFortessa X-20 flow cytometer (BD Biosciences). The data were processed with FlowJo (Treestar).

### Human cytokine antibody array

Cancer cells were cultured in a serum-free medium, and after 24 h, the supernatant was collected and filtered to prepare a conditioned medium. The cytokines in the cell medium were examined by a human cytokine antibody array (Raybiotech) according to the manufacturer’s protocol. Briefly, membranes precoated with the indicated antibodies were sequentially incubated with coating buffer, blocking buffer, conditioned medium samples, biotin-labeled antibody, and Cy3-labeled streptavidin. The membranes were then scanned with a GenePix 4000B system (Axon Instruments). The fluorescence signals were processed using GenePix Pro 6.0 software (Axon Instruments), and the values of each cytokine were normalized to their positive control values.

### Enzyme-linked immunosorbent assay (ELISA)

The concentrations of human MIF in conditioned media or in orthotopic tumors were detected using “sandwich” ELISA kits (Multi Sciences) according to the manufacturer’s protocol. Briefly, serum-free supernatants were harvested after 24 h of cell culture, and the tumor lysates were prepared as described previously^22^. The cell supernatants, tumor lysates, or standard samples were added to wells precoated with the indicated antibodies and then incubated at room temperature for 2 h. The wells were sequentially incubated with detecting antibodies, HRP-labeled streptavidin, substrate solution, and stop solution. The absorbance was measured with a multimode microplate reader (Molecular Devices), and the final concentration of each protein was calculated according to its standard curve.

### Statistical analysis

All comparisons between two variables were analyzed by a two-tailed Student’s *t* test. Comparisons between two Kaplan‒Meier curves were analyzed by the long-rank test, and the optimal cutoff was defined as described previously^23^. Forest plots showing the multivariable analysis of prognostic parameters for overall survival were determined using a multivariate Cox regression model. Correlations between two variables were assessed by Pearson correlation analysis. A *P* value lower than 0.05 was considered to indicate statistical significance.

## Results

### MAPK4 downregulation is associated with liver metastasis and poor prognosis

**Fig. 1:**
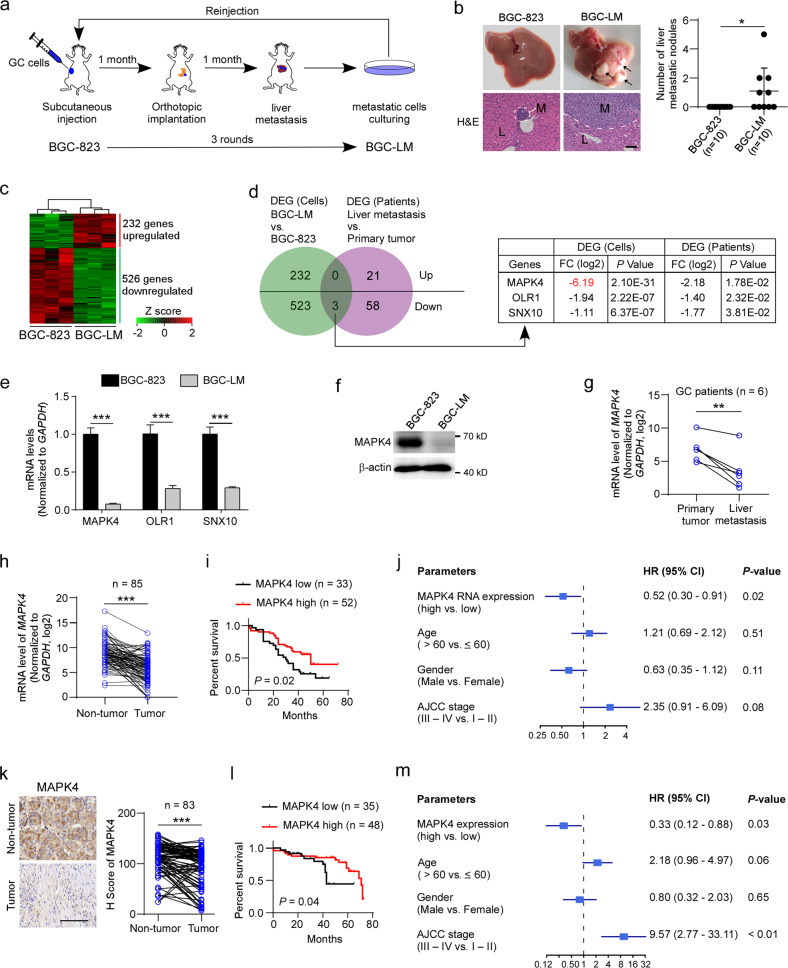
MAPK4 downregulation is associated with liver metastasis and poor prognosis in gastric cancer patients. **a** Schematic of in vivo selection for the isolation of highly metastatic gastric cancer cells using an orthotopic xenograft mouse model in SCID mice. **b** BGC-823 and BGC-LM cells were subjected to orthotopic implantation, and the number of liver metastatic nodules per mouse was counted. Representative photographs of livers or hematoxylin and eosin (H&E) staining of liver sections are shown. The arrows denote liver metastatic nodules. Scale bar 100 μm. L liver tissue, M metastasis. **c** Heatmap representing the differential gene expression patterns between BGC-823 and BGC-LM cells (|fold change, log2 | ≥ 1 and *p* < 0.05 by Student’s *t* test). **d** Venn diagram showing the overlap of genes associated with gastric cancer liver metastasis. Differentially expressed genes (DEGs) of human liver metastatic tissues compared with their matched primary tumor tissues from a previously reported cohort (GSE11072) and DEGs of BGC-LM cells compared with BGC-823 cells are shown. FC fold change. **e** Analysis of the expression levels of MAPK4, OLR1, and SNX10 in BGC-823 and BGC-LM cells by quantitative RT-PCR. MAPK4 mitogen-activated protein kinase 4, OLR1 oxidized low-density lipoprotein receptor 1, SNX10 sorting nexin 10. **f** Analysis of MAPK4 expression levels in BGC-823 and BGC-LM cells by western blotting. **g** Quantitative RT-PCR analysis of *MAPK4* mRNA in tumor tissues and matched liver metastatic lesions from gastric cancer patients. **h**, **k** MAPK4 expression levels in gastric cancer tissues and paired adjacent nontumor tissues determined by qRT-PCR (**h**) or immunohistochemistry (**k**). Representative photographs of MAPK4 staining are shown. Scale bar, 100 μm. **i**, **l** Kaplan‒Meier analysis of the correlation between the MAPK4 levels in gastric cancer tissues and overall survival. **j**, **m** Multivariable analysis of prognostic factors for gastric cancer patients. AJCC American Joint Committee on Cancer, CI confidence interval, HR hazard ratio, GC gastric cancer. The data are expressed as the means ± standard deviations. *p < 0.05, **p < 0.01, ***p < 0.001 (Student’s *t* test).

To study the mechanism of gastric cancer liver metastasis, we selected highly metastatic cells in vivo using an orthotopic xenograft mouse model. In detail, we implanted a subcutaneous tumor piece from the human gastric cancer cell line BGC-823 on the stomach wall of SCID mice to establish an orthotopic model of gastric cancer cells (Fig. 1a). We then isolated metastatic cells from the liver for expansion via in vitro cell culture, and these expanded cells were then implanted into SCID mice for a second round of in vivo selection. A highly metastatic population of BGC-823 cells, which was denoted BGC-LM, was obtained after three rounds of selection. BGC-LM cells developed macroscopic metastatic nodules (namely, macrometastases) in the liver in half of the SCID mice, whereas the original BGC-823 cells failed to induce macrometastases in any of the SCID mice (Fig. 1b), which indicated that BGC-LM cells exhibit a higher metastatic capacity than their parental BGC-823 cells.

To explore the genes involved in the liver metastasis of gastric cancer, we systematically compared the transcriptome profiles between BGC-823 and BGC-LM cells by RNA sequencing (Fig. 1c). The data showed that a total of 758 genes were significantly differentially expressed in BGC-LM cells compared with BGC-823 cells, and these included 232 upregulated genes and 526 downregulated genes. To determine the key genes associated with gastric cancer liver metastasis clinically, we analyzed a previously reported dataset (GSE11072) from a cohort containing liver metastatic tissues and matched primary tumor tissues from gastric cancer patients (Fig. 1d). Further Venn diagrams revealed three overlapping genes that were downregulated in these metastatic cells and tissues. Among these genes, MAPK4 was the most significantly downregulated gene, as was further confirmed by quantitative real-time PCR (qRT-PCR) assay (Fig. 1e). Moreover, we confirmed that MAPK4 expression was robustly decreased in BGC-LM cells compared with BGC-823 cells by western blotting (Fig. 1f), and this finding was verified by reduced MAPK4 expression in liver metastatic tissues compared with matched primary tumor tissues from gastric cancer patients (Fig. 1g). These data suggest that MAPK4 downregulation is associated with gastric cancer liver metastasis.

To investigate the clinical significance of MAPK4 in gastric cancer progression, we examined *MAPK4* mRNA expression in paired tumor and nontumor tissues from gastric cancer patients by qRT-PCR. The results showed that gastric cancer tissues had significantly lower expression levels of *MAPK4* than their matched adjacent nontumor tissues (Fig. 1h). Kaplan‒Meier analysis revealed that lower expression of *MAPK4* in gastric cancer tissues was significantly associated with reduced overall survival time of patients (Fig. 1i). Multivariate analysis showed that *MAPK4* expression in tumor tissues was an independent factor for predicting the prognosis of gastric cancer patients (Fig. 1j). Moreover, we evaluated MAPK4 protein expression using a gastric cancer tissue array containing tumor tissues and paired nontumor tissues from gastric cancer patients. Consistently, MAPK4 protein was also significantly decreased in gastric cancer tissues (Fig. 1k). MAPK4 downregulation appeared to be an independent risk factor for poor prognosis in patients with gastric cancer (Fig. 1l, m). Thus, these findings indicate that the downregulation of MAPK4 in gastric cancer tissues is associated with tumor progression and poor prognosis in gastric cancer patients.

### The depletion of MAPK4 promotes liver metastasis of gastric cancer cells

**Fig. 2:**
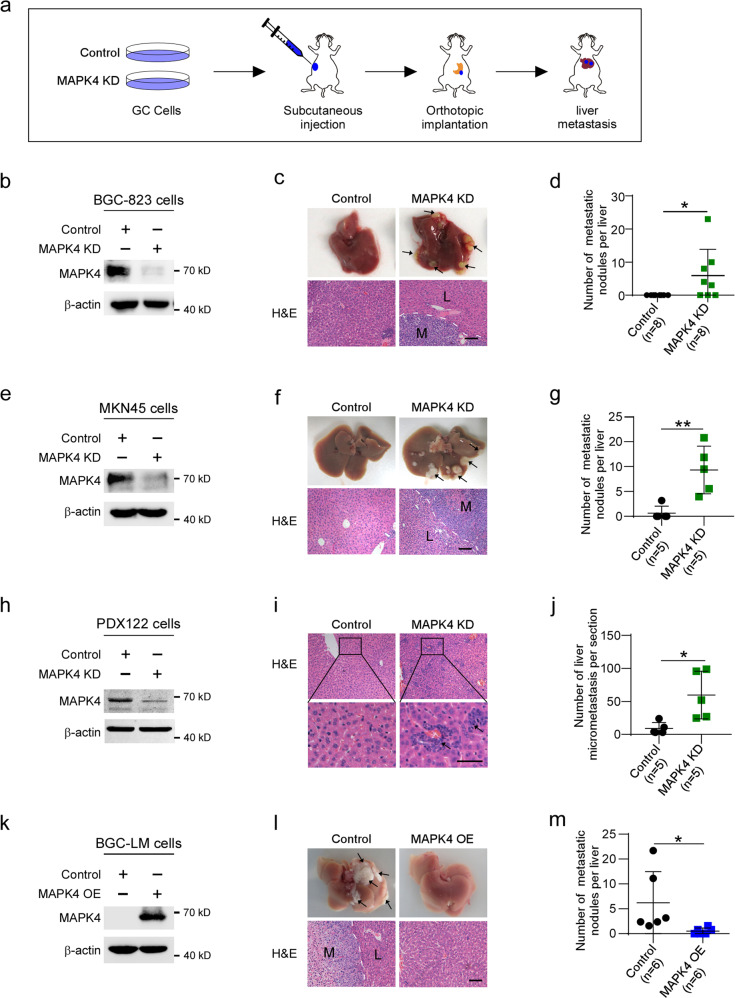
MAPK4 downregulation promotes gastric cancer liver metastasis in orthotopic mouse models. **a** Schematic illustration of orthotopic implantation to develop liver metastasis using gastric cancer cells in SCID mice. **b**–**d** BGC-823 cells were treated with lentivirus-based shRNA targeting MAPK4 and subjected to western blotting with the indicated antibodies and orthotopic implantation. **e**–**g** MKN45 cells infected with shRNA targeting MAPK4 were used for western blotting and orthotopic implantation. **h**–**j** PDX122 cells were treated with shRNA targeting MAPK4 and subjected to western blotting and orthotopic implantation. **k**–**m** BGC-LM cells infected with pLVX-MAPK4 lentivirus were used for western blotting and orthotopic implantation. Representative photographs of livers or H&E staining of the liver sections are shown. The arrows denote liver metastatic nodules (**c**, **f**, **l**) or liver micrometastases (**i**). Scale bars, 100 μm (**c**, **f**, **l**) or 50 μm (**i**). Quantitative analysis of liver metastatic nodules per mouse (**d**, **g**, **m**) or liver micrometastases per section (**j**). KD knockdown, OE overexpression. The data are expressed as the means ± standard deviations. *p < 0.05, **p < 0.01, ***p < 0.001 (Student’s *t* test).

To determine whether MAPK4 downregulation promotes gastric cancer liver metastasis in orthotopic mouse models, we employed a lentivirus-based short-hairpin RNA (shRNA) to stably deplete MAPK4 expression in human gastric cancer cell lines, including BGC-823 and MKN45 cells (Fig. 2a). The results revealed that more than 60% of SCID mice in the MAPK4-inhibited group developed liver metastatic nodules, whereas all of the SCID mice in the control group remained free of macrometastases (Fig. 2b–g). Because cells from patient-derived xenografts are more similar than immortal cell lines to tumors^24^, we developed a cell line (PDX122) directly from gastric cancer patient-derived xenograft^25^. The knockdown of MAPK4 in PDX122 cells also significantly increased the number of liver micrometastatic lesions in an orthotopic mouse model (Fig. 2h–j). In agreement with the above-described data, the overexpression of MAPK4 significantly decreased the liver metastatic capacity of BGC-LM cells (Fig. 2k–m). Collectively, these observations indicate that MAPK4 negatively regulates gastric cancer liver metastasis.

### MAPK4 downregulation promotes gastric cancer cell invasion in vivo

To explore the mechanism through which MAPK4 downregulation promotes liver metastasis of gastric cancer, we tested whether MAPK4 influences the proliferation of gastric cancer cells. Our results showed that the depletion of MAPK4 had no significant effects on the proliferation or colony formation of BGC-823 and MKN45 cells (Supplementary Fig. 1a–f) or on the tumor growth of BGC-823 cells (Supplementary Fig. 2a–c). The ectopic expression of MAPK4 in BGC-LM cells also yielded similar results (Supplementary Figs. 1g–i and 2d–f). We then examined the role of MAPK4 in gastric cancer cell invasion. Our data revealed that the depletion of MAPK4 suppressed cell invasion (Supplementary Fig. 3a, b). These results conflict with the in vivo phenotypes in which the knockdown of MAPK4 in BGC-823 cells promoted liver metastasis (Fig. 2b–d).

To resolve the paradox of the inconsistent functions of MAPK4 in vivo and in vitro, we explored the possibility that the tumor microenvironment is involved in MAPK4 downregulation-induced invasion. We educated BGC-823 cells depleted of MAPK4 in orthotopic mouse models and isolated gastric cancer cells from orthotopic tumors as described previously (Supplementary Fig. 3c)^26^. After education, MAPK4-depleted cells had a significantly higher capacity for cell invasion than control cells (Supplementary Fig. 3d, e). Consistently, MAPK4-overexpressing BGC-LM cells educated by the tumor microenvironment had a reduced capacity for cell invasion compared with control cells (Supplementary Fig. 3f, g). Taken together, these results suggest that the tumor microenvironment may be essential for MAPK4 downregulation-induced gastric cancer cell invasion.

### MAPK4 depletion polarizes TAMs to promote gastric cancer cell invasion

**Fig. 3:**
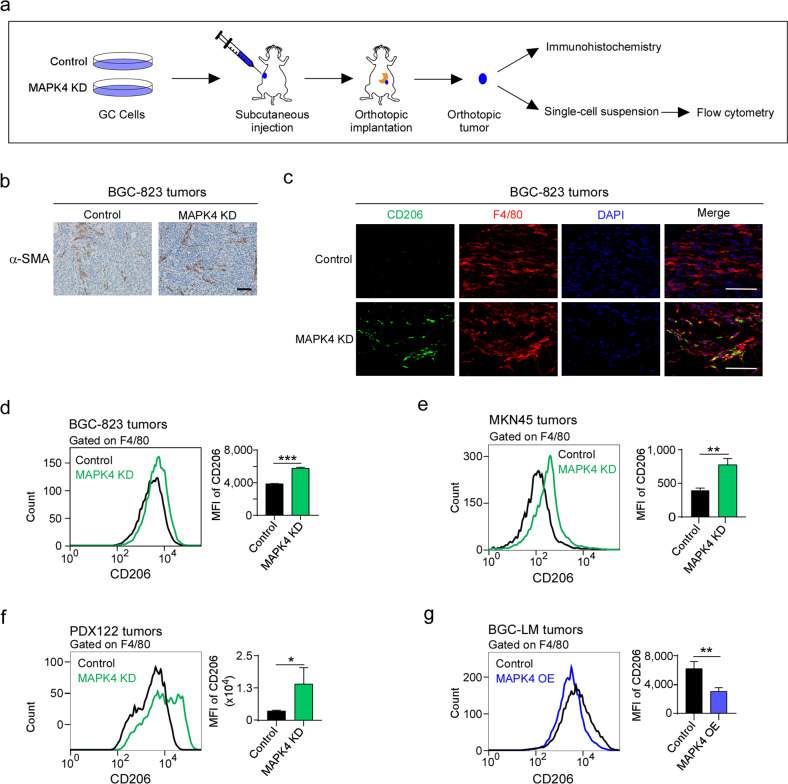
MAPK4 downregulation in gastric cancer cells polarizes TAMs in orthotopic tumors. **a** Schematic representation of orthotopic gastric cancer tumors for immunohistochemistry or flow cytometry analysis. **b** Orthotopic tumors of MAPK4-depleted BGC-823 cells or controls were subjected to immunohistochemistry with anti-α-SMA antibody (brown). The nuclei were stained with hematoxylin (blue). Scale bars, 100 μm. **c** Control and MAPK4-depleted BGC-823 cells were used for orthotopic implantation. Orthotopic tumors were further analyzed by immunofluorescence with anti-F4/80 (red) and CD206 (green) antibodies. The nuclei were stained with DAPI (blue). Scale bars, 50 μm. **d**–**f** Single-cell suspensions were prepared from orthotopic tumors of BGC-823 (**d**), MKN45 (**e**) or PDX122 (**f**) cells depleted or not depleted of MAPK4 and analyzed by flow cytometry with anti-F4/80 and anti-CD206 antibodies. Quantification of CD206 expression in F4/80-positive cells is shown. MFI mean fluorescence intensity. The gating of F4/80-positive cells is shown in Supplementary Fig. 4a–c. **g** Single-cell suspensions from orthotopic tumors of control and MAPK4-overexpressing BGC-LM cells were stained with F4/80 and CD206. CD206 expression in F4/80-positive cells was analyzed by flow cytometry. The gating of F4/80-positive cells is shown in Supplementary Fig. 4d. The data are expressed as the means ± standard deviations. *p < 0.05, **p < 0.01, ***p < 0.001; NS, not significant (Student’s *t* test).

It is widely recognized that activated stromal compartments, including cancer-associated fibroblasts (CAFs) and TAMs, play crucial roles in metastasis, including gastric cancer metastasis^27–29^. To investigate whether MAPK4 downregulation promotes gastric cancer metastasis by activating stromal cells, we examined whether MAPK4 knockdown affects the abundance of CAFs in orthotopic tumors of gastric cancer cells (Fig. 3a). An immunochemistry analysis of MAPK4-depleted tumors compared with control tumors showed no significant difference in the expression of α-SMA, a marker of cancer-associated fibroblasts (Fig. 3b).

Because protumor TAMs in gastric cancer tissues typically exhibit high levels of CD206 expression^10,30,31^, we tested whether MAPK4 knockdown affects CD206 expression in macrophages in an orthotopic tumor model. Our data revealed that the depletion of MAPK4 increased the percentage of CD206-positive cells among total macrophages positive for F4/80 (Fig. 3c). To confirm the role of MAPK4 in the regulation of TAM activation, we used flow cytometry to gate the total macrophage population from orthotopic tumor tissues by F4/80 staining and examined CD206 expression in this population. The results showed that the knockdown of MAPK4 in BGC-823, MKN45, and PDX122 cells significantly increased CD206 expression in F4/80-positive macrophages without affecting the percentage of macrophages in tumors (Fig. 3d–f and Supplementary Fig. 4a–c). In addition, the ectopic expression of MAPK4 significantly reduced the expression of CD206 in macrophages from orthotopic tumors of BGC-LM cells (Fig. 3g and Supplementary Fig. 4d). Thus, these findings highlight that MAPK4 depletion in gastric cancer cells polarizes TAMs in an orthotopic tumor model.

**Fig. 4:**
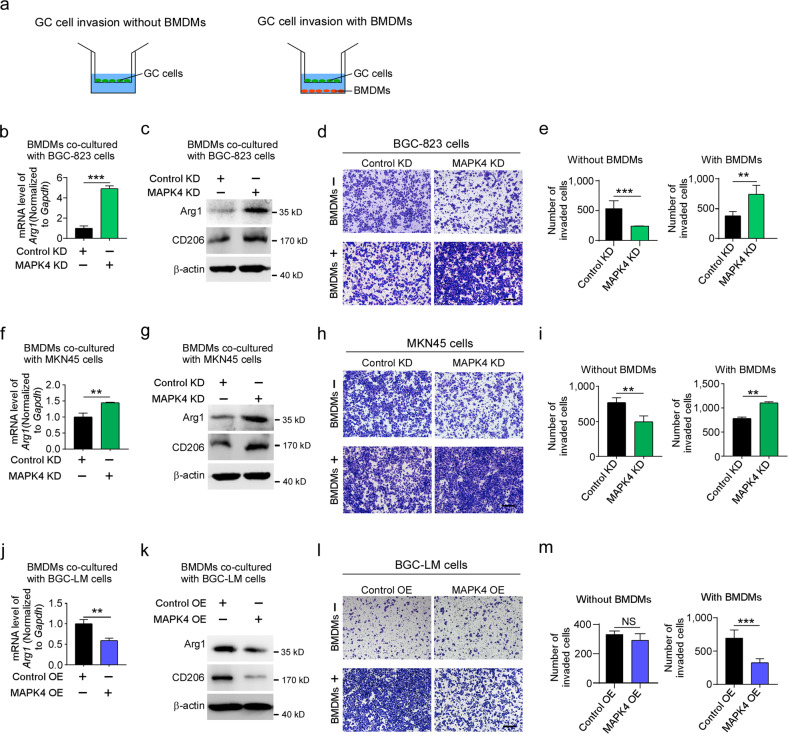
MAPK4 downregulation promotes TAM polarization and cancer cell invasion in a coculture system. **a** Schematic of the coculture of gastric cancer cells with or without BMDMs in the Boyden chamber system. The upper chamber was coated with Matrigel. **b**, **c** Quantitative RT-PCR analysis of *Arg1* mRNA and western blot analysis of Arg1 and CD206 proteins in BMDMs cocultured with control and MAPK4-depleted BGC-823 cells. **d**, **e** Control and MAPK4-depleted BGC-823 cells cocultured or not cocultured with BMDMs were subjected to cell invasion analysis. The quantification of invaded cells per well is shown. **f**, **g** Quantitative RT‒PCR analysis of *Arg1* mRNA and western blot analysis of Arg1 and CD206 proteins in BMDMs cocultured with control and MAPK4-depleted MKN45 cells. **h**, **i** Control and MAPK4-depleted MKN45 cells cocultured or not cocultured with BMDMs was subjected to cell invasion analysis. The number of invaded cells per well was quantified. **j**, **k** Quantitative RT-PCR analysis of *Arg1* mRNA and western blot analysis of Arg1 and CD206 proteins in BMDMs cocultured with control and MAPK4-overexpressing BGC-LM cells. **l**, **m** MAPK4-overexpressing BGC-LM cells cocultured or not cocultured with BMDMs were used for the cell invasion assay. The number of invaded cells per well was quantified. Scale bar, 200 μm. The data are expressed as the means ± standard deviations. **p < 0.01, ***p < 0.001; NS not significant (Student’s *t* test).

To determine whether macrophages mediate the function of MAPK4 in gastric cancer development, we generated a coculture model of gastric cancer cells and bone marrow-derived macrophages (BMDMs) (Fig. 4a). In this system, the depletion of MAPK4 in BGC-823 cells and MKN45 cells not only significantly enhanced the expression of arginase 1 (Arg1, a marker for activated TAM) and CD206 in BMDMs but also robustly promoted gastric cancer cell invasion (Fig. 4b–i). Moreover, forced expression of MAPK4 in BGC-LM cells significantly inhibited macrophage activation and gastric cancer cell invasion in this coculture model (Fig. 4j–m). Together, these results indicate that MAPK4 downregulation in gastric cancer cells promotes their invasion by activating TAMs.

### MAPK4 downregulation increases MIF secretion to activate TAMs

**Fig. 5:**
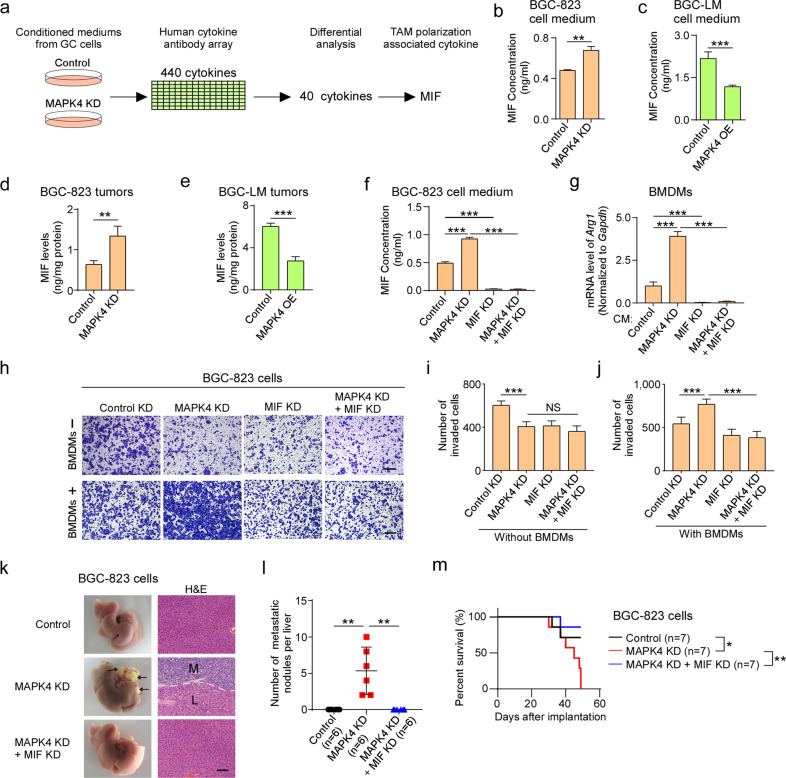
MAPK4 downregulation increases MIF secretion to activate TAMs. **a** Screening of cytokines to promote TAM polarization from conditioned media of control and MAPK4-depleted BGC-823 cells. **b** Quantitative analysis of the MIF levels in conditioned media from control and MAPK4-depleted BGC-823 cells by ELISA. **c** Quantification of the MIF levels in conditioned media from control and MAPK4-overexpressing BGC-LM cells by ELISA. **d** Quantitative analysis of the MIF levels in orthotopic tumors of BGC-823 cells depleted of MAPK4 by ELISA. **e** Quantification of the MIF levels in orthotopic tumors of BGC-LM cells overexpressing MAPK4 by ELISA. **f** Analysis of the MIF levels in conditioned media from BGC-823 cells with the indicated treatments by ELISA. **g** Quantitative RT-PCR analysis of *Arg1* mRNA in BMDMs cocultured with BGC-823 cells with the indicated treatments. **h**–**j** BGC-823 cells with the indicated treatments were used for Matrigel invasion analysis. The number of invaded cells per well was quantified. Scale bars, 200 μm. **k**–**m** BGC-823 cells were depleted of the indicated genes and subjected to orthotopic implantation to induce the development of liver metastasis in SCID mice. Representative photographs of livers and H&E staining of the liver sections are shown (**k**). The metastatic nodules per mouse were counted (**l**). The arrows denote metastatic nodules. Scale bars, 100 μm. The indicated mice were also subjected to Kaplan‒Meier analysis (log-rank test) (**m**). The data are expressed as the means ± standard deviations. *p < 0.05, **p < 0.01, ***p < 0.001; NS not significant (Student’s *t* test).

Given that TAMs are generally activated by extracellular cytokines within tumors^32–34^, we compared the cytokine secretion profiles of MAPK4-depleted BGC-823 cells with those of control cells to explore how MAPK4 downregulation in gastric cancer cells activates TAMs. A human cytokine antibody array showed that 40 cytokines were differentially secreted after MAPK4 depletion (fold change > 1.5) (Fig. 5a and Supplementary Table 1). Among these, 14 cytokines were upregulated in the MAPK4-depleted group and are hypothesized to be potential inducers of M2 macrophages. Because MIF has been reported to induce M2-macrophage polarization^35^, we chose to focus on MIF for further studies. ELISA confirmed that MIF secretion was significantly increased after the depletion of MAPK4 in BGC-823 cells (Fig. 5b). Moreover, the ectopic expression of MAPK4 in BGC-LM cells also significantly decreased MIF secretion (Fig. 5c). Together, these results indicate that MIF may be a TAM-associated cytokine induced by MAPK4 downregulation in BGC-823 cells.

To investigate whether MAPK4 inhibits MIF in vivo, we analyzed the MIF levels in orthotopic tumors by ELISA. The data revealed that the depletion of MAPK4 significantly increased the MIF levels in orthotopic tumors of BGC-823 cells (Fig. 5d), whereas MAPK4 overexpression significantly decreased the MIF levels in orthotopic tumors of BGC-LM cells (Fig. 5e). These findings suggest that MAPK4 downregulation in gastric cancer cells enhances the secretion of MIF.

To further investigate whether MIF is responsible for TAM polarization and gastric cancer cell invasion induced by MAPK4 downregulation, we first examined MIF expression and TAM polarization in an orthotopic tumor model. Our data showed that the depletion of MAPK4 significantly increased the expression of MIF in gastric cancer cells and the percentage of CD206-positive macrophages in orthotopic tumors (Supplementary Fig. 5). In addition, we depleted MIF in MAPK4-depleted BGC-823 cells in a coculture system of cancer cells and BMDMs (Fig. 4a). In this coculture system, the downregulation of MIF in MAPK4-depleted BGC-823 cells not only robustly inhibited MIF secretion but also significantly suppressed *Arg1* expression in BMDMs and gastric cancer cell invasion (Fig. 5f–j). Furthermore, in the orthotopic mouse model of MAPK4-depleted BGC-823 cells, the knockdown of MIF significantly suppressed liver metastasis and prolonged the survival time of SCID mice (Fig. 5k–m). Taken together, these results imply that the downregulation of MAPK4 in gastric cancer cells enhances MIF secretion to activate TAMs, which increases gastric cancer cell invasion and liver metastasis.

### MAPK4 promotes MIF degradation in gastric cancer cells

**Fig. 6:**
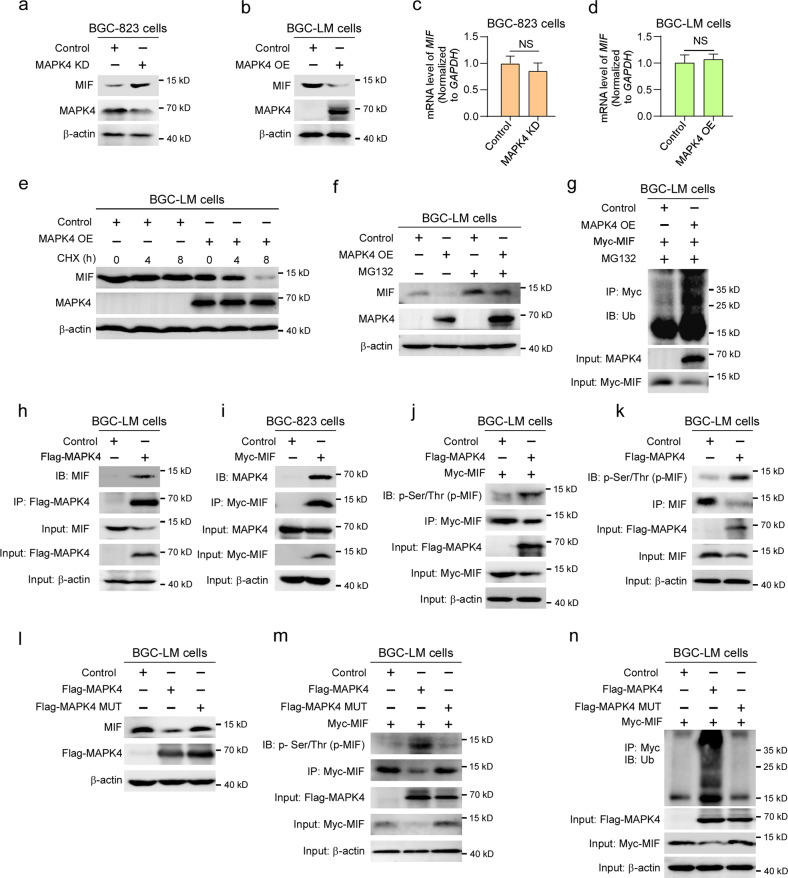
MAPK4 promotes MIF degradation in gastric cancer cells. **a** Western blot analysis of MIF in control and MAPK4-depleted BGC-823 cells. **b** Western blot analysis of MIF in control and MAPK4-overexpressing BGC-LM cells. **c** Quantitative RT-PCR analysis of *MIF* mRNA in control and MAPK4-depleted BGC-823 cells. **d** Quantitative RT-PCR analysis of *MIF* mRNA in control and MAPK4-overexpressing BGC-LM cells. **e**, **f** BGC-LM cells, infected or not infected with pLVX-MAPK4 lentivirus were treated with cycloheximide for the indicated times or MG132 and used for western blot analysis. **g** Control and MAPK4-overexpressing BGC-LM cells were transfected with the Myc-MIF vector, treated with MG132, and subjected to immunoprecipitation analysis with anti-Myc antibody. **h** BGC-LM cells transfected with control or Flag-MAPK4 vector were used for immunoprecipitation analysis with anti-FLAG antibody. **i** BGC-823 cells were transfected with control or Myc-MIF vector and subjected to immunoprecipitation analysis with anti-Myc antibody. **j** BGC-LM cells transfected with the indicated vectors were subjected to immunoprecipitation analysis with anti-Myc antibody. **k** BGC-LM cells were transfected or not transfected with Flag-MAPK4 and subjected to immunoprecipitation analysis with an anti-MIF antibody. **l** BGC-LM cells transfected with the indicated vectors were used for western blotting. **m**, **n** BGC-LM cells were transfected with the indicated vectors and subjected to immunoprecipitation analysis with anti-Myc antibody. The data are expressed as the means ± standard deviations. NS not significant (Student’s *t* test).

To determine the mechanism through which MAPK4 inhibits MIF secretion in gastric cancer cells, we examined the effects of MAPK4 expression on the protein or mRNA levels of MIF. Western blotting showed that the knockdown of MAPK4 increased the MIF levels in BGC-823 cells, whereas the ectopic expression of MAPK4 reduced the MIF levels in BGC-LM cells (Fig. 6a, b). Quantitative RT‒PCR revealed that the *MIF* mRNA levels remained invariable regardless of the knockdown or ectopic expression of MAPK4 in gastric cancer cells (Fig. 6c, d). Furthermore, the protein levels of secretory and intracellular MIF were significantly higher in BGC-LM cells than in BGC-823 cells, but the mRNA levels of *MIF* were comparable (Supplementary Fig. 6). These results suggest that MIF expression may be regulated by MAPK4 at the posttranscriptional level. To test this hypothesis, we treated BGC-LM cells with the protein synthesis inhibitor cycloheximide and found that the ectopic expression of MAPK4 clearly increased the degradation of MIF (Fig. 6e).

Because many cellular proteins are degraded through the ubiquitin‒proteasome system^36^, we investigated whether MAPK4 influences the ubiquitination of MIF. The results revealed that the proteasome inhibitor MG-132 effectively increased the protein levels of MIF in BGC-LM cells with exogenous expression of MAPK4 (Fig. 6f). An immunoprecipitation analysis showed that the ectopic expression of MAPK4 markedly increased the ubiquitination of MIF in BGC-LM cells in the presence of MG132 (Fig. 6g). These data indicate that MAPK4 may promote the ubiquitination-dependent degradation of MIF in gastric cancer cells.

Accumulating evidence shows that MAPK-mediated phosphorylation enhances the ubiquitination of their substrates^37,38^. To examine whether MAPK4-mediated phosphorylation increases the ubiquitination of MIF, we first tested the interaction between MAPK4 and MIF by coimmunoprecipitation analysis and found that exogenous MAPK4 bound to endogenous MIF in BGC-LM cells (Fig. 6h). Accordingly, ectopic Myc-MIF also showed an interaction with endogenous MAPK4 (Fig. 6i). Furthermore, the overexpression of MAPK4 not only obviously increased the phosphorylation of MIF but also reduced the levels of MIF (Fig. 6j, k). To investigate whether MAPK4-induced MIF ubiquitination depends on the catalytic activity of MAPK4, we generated a kinase-dead mutant of MAPK4 (K49A and K50A) as described previously^39^. The results revealed that the kinase-dead mutation of MAPK4 not only restored the MIF protein levels but also reduced the phosphorylation and ubiquitination of MIF (Fig. 6l–n). Taken together, these data imply that MAPK4 may phosphorylate MIF to promote its ubiquitination, which subsequently induces MIF degradation.

### TAM-induced EMT inhibits MAPK4 expression in gastric cancer cells

**Fig. 7:**
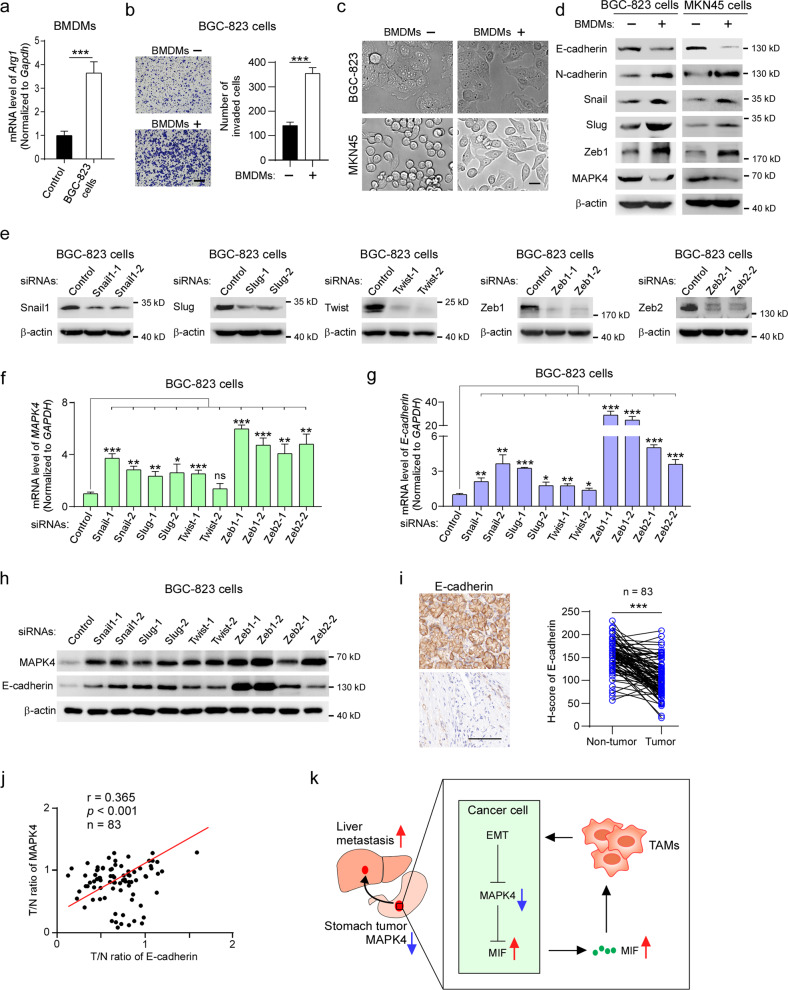
TAM-induced EMT inhibits MAPK4 expression in gastric cancer cells. **a** BMDMs were cocultured or not cocultured with BGC-823 cells and subjected to qRT‒PCR analysis of *Arg1* mRNA. **b** BGC-823 cells were cocultured or not cocultured with BMDMs and were used for the cell invasion assay. Scale bar, 200 μm. **c**, **d** Cell morphology and western blot analyses of BGC-823 and MKN45 cells cocultured or not cocultured with BMDMs. Scale bar, 20 μm. **e** BGC-823 cells were transfected with the indicated siRNAs and subjected to western blotting with the indicated antibodies. **f**, **g** Quantitative RT-PCR analysis of MAPK4 and E-cadherin in BGC-823 cells transfected with the indicated siRNAs. **h** BGC-823 cells transfected with the indicated siRNAs were subjected to western blotting. **i** Immunohistochemical analysis of E-cadherin in a human gastric tumor tissue array. Representative photographs of immunohistochemical staining in gastric cancer tissues (lower left) and adjacent nontumor tissues (upper left) are shown. Scale bar, 100 μm. **j** Pearson correlation analysis of relative MAPK4 expression (tumor/nontumor) from Fig. 1k and relative E-cadherin expression (tumor/nontumor) from (**i**). **k** Working model of MAPK4 downregulation-mediated positive feedback between cancer cells and macrophages to facilitate gastric cancer liver metastasis. The data are expressed as the means ± standard deviations. *p < 0.05, **p < 0.01, ***p < 0.001; NS not significant (Student’s *t* test).

Given that EMT is a key process enabling cancer cells to acquire the capacities of invasion and metastasis, which can be activated by stromal cells, including macrophages^12,40–43^, we attempted to investigate whether TAMs promote the EMT process of gastric cancer cells. Our results showed that BMDMs cocultured with BGC-823 cells exhibited higher *Arg1* expression than control BMDMs (Fig. 7a). Importantly, BGC-823 cells cocultured with BMDMs exhibited a stronger cell invasion capacity (Fig. 7b). After coculture with BMDMs, BGC-823 and MKN45 cells showed obvious mesenchymal-like morphology (Fig. 7c). Moreover, we also detected decreased expression of E-cadherin and increased expression of N-cadherin, Snail, Slug and Zeb1 in both cell lines after coculture with BMDMs (Fig. 7d), which implies that TAMs may activate EMT of gastric cancer cells.

To study how MAPK4 is downregulated in gastric cancer cells, we investigated whether TAMs inhibit MAPK4 expression. Our data showed that coculture with BMDMs not only induced EMT in BGC-823 and MKN45 cells but also significantly suppressed the expression of MAPK4 in BGC-823 and MKN45 cells (Fig. 7d), which indicated that TAM-induced EMT may be involved in the inhibition of MAPK4 expression. To confirm this hypothesis, we knocked down the key transcription factors of EMT using small interfering RNAs and found that the inhibition of most EMT transcription factors significantly increased the mRNA and protein expression of MAPK4 and E-cadherin (Fig. 7e–h). Taken together, these data suggest that TAMs activate EMT in gastric cancer cells to inhibit MAPK4 expression.

### MAPK4 expression is associated with E-cadherin in gastric cancer tissues

To further explore the association of MAPK4 expression and EMT in gastric cancer tumors of patients, we employed tissue array analysis. The results showed that E-cadherin was significantly downregulated in tumor tissues compared with adjacent nontumor tissues and appeared to be an independent predictive factor of poor prognosis in gastric cancer patients (Fig. 7i and Supplementary Fig. 7), which was in agreement with our previous data on MAPK4 expression (Fig. 1k–m). Furthermore, the relative levels of MAPK4 were significantly correlated with those of E-cadherin in gastric cancer tissues from patients (Fig. 7j). Thus, these observations imply that MAPK4 expression is associated with EMT in gastric cancer tissues.

## Discussion

In this study, we found that MAPK4 downregulation in gastric cancer tissues from patients is significantly associated with liver metastasis and poor prognosis. In orthotopic mouse models, MAPK4 downregulation in gastric cancer cells increases MIF secretion to activate TAMs, which activates cancer cell EMT to further inhibit MAPK4 expression, and these findings suggest a positive feedback loop between gastric cancer cells and TAMs to drive liver metastasis (Fig. 7k).

It is well known that TAM infiltration is associated with the progression and metastasis of cancers, including gastric cancer, in clinical settings^7,10,11^. Previous studies have shown that TAMs promote cancer metastasis by interacting with cancer cells^12,44,45^. However, the role of the interaction between cancer cells and macrophages in gastric cancer metastasis remains poorly understood. Here, we provide evidence showing that MAPK4 downregulation mediates a previously unrecognized positive feedback loop between cancer cells and TAMs in gastric cancer, which constitutes a “vicious cycle” to drive gastric cancer liver metastasis.

MIF, which was first discovered to inhibit the migration of macrophages in vitro, plays crucial roles in innate immune and inflammatory responses^46,47^. Recently, MIF expression has been documented to be upregulated in many cancers^48,49^ and to promote breast cancer and melanoma metastasis by inducing myeloid-derived suppressor cells and TAMs, respectively^35,50,51^. Nevertheless, the mechanism of MIF stability regulation and the role of MIF in gastric cancer metastasis remain unclear. In this study, we found that MAPK4 interacts with and phosphorylates MIF to promote its ubiquitination and degradation in gastric cancer cells. The downregulation of MAPK4 in gastric cancer cells increases MIF secretion to promote macrophage polarization, which facilitates gastric cancer liver metastasis.

MAPK4 is an atypical MAPK that lacks the conserved Thr-X-Tyr activation motif^39,52^. *Mapk4*-deficient mice are viable and fertile and exhibit no gross morphological or physiological anomalies^53^. A bioinformatics analysis of The Cancer Genome Atlas revealed that MAPK4 upregulation is significantly correlated with decreased overall survival in patients with lung adenocarcinoma, bladder cancer, low-grade glioma, and thyroid carcinoma^54^. The overexpression of MAPK4 has been reported to promote tumor progression via noncanonical activation of AKT/mTOR signaling^54^. However, the role of MAPK4 in cancer metastasis remains unclear. Our data show that MAPK4 downregulation in gastric cancer tissues is significantly associated with liver metastasis and poor prognosis in patients. The depletion of MAPK4 facilitates the invasion and metastasis of gastric cancer cells by polarizing TAMs in orthotopic mouse models. The forced expression of MAPK4 significantly decreases the liver metastatic capacity of gastric cancer cells. These findings indicate a hitherto unrecognized role of MAPK4 as a metastasis suppressor in gastric cancer.

## Supplementary information

Supplemental Figures, table and methods
