# Supplementary material for: MAPK4 silencing in gastric cancer drives liver metastasis by positive feedback between cancer cells and macrophages
Source: Exp Mol Med. 2023 Feb 17;55(2):457–69. doi: 10.1038/s12276-023-00946-w (PMC9981715; doi:10.1038/s12276-023-00946-w)
Supplement: Supplementary file 1 — Supplemental Figures, table and methods [file 12276_2023_946_MOESM1_ESM.pdf]

Supplemental Figures

Supplementary Fig. 1

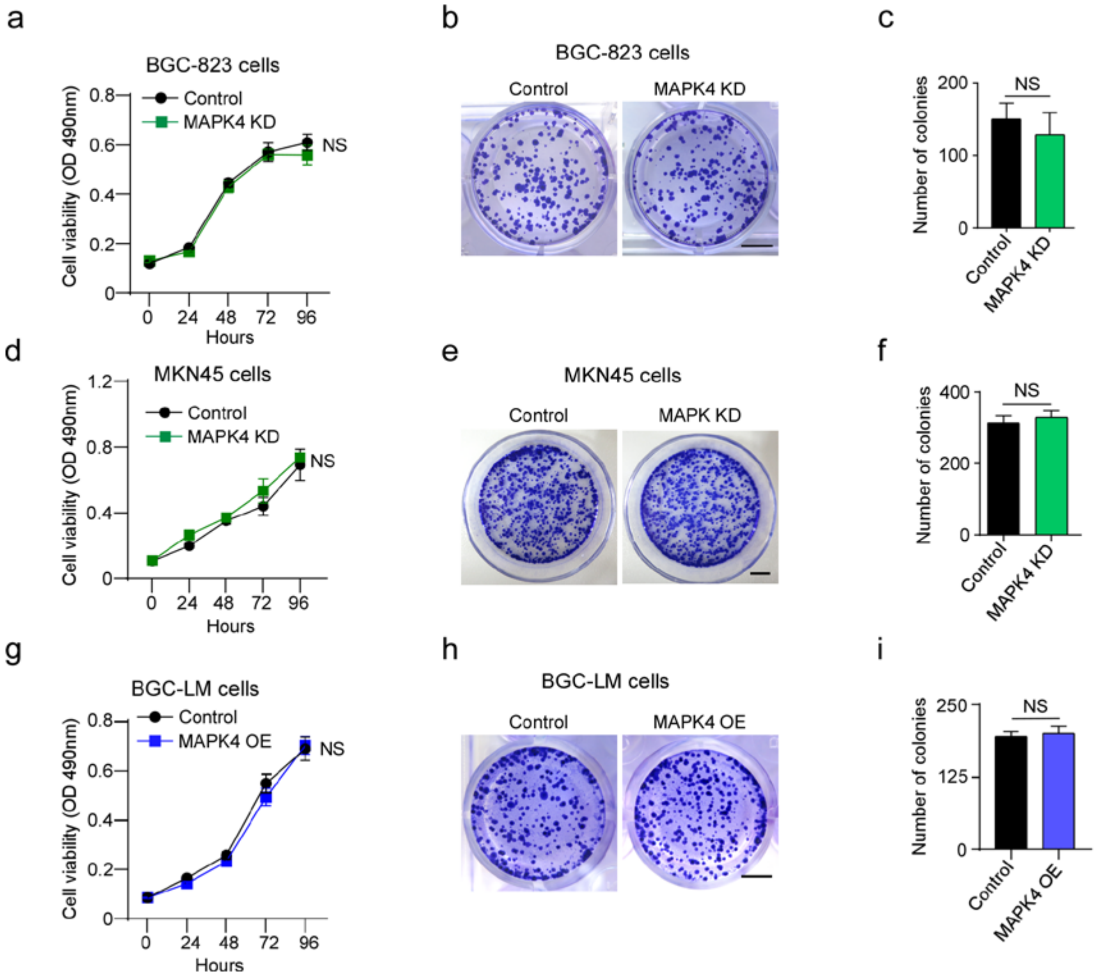

Supplementary Fig. 1. MAPK4 has no significant effect on the proliferation of gastric cancer cells.

(a-c) BGC-823 cells were treated with the lentivirus-based shRNA targeting MAPK4 and subjected to MTT and colony formation analyses. Cell colonies per well were quantified. Scale bar, 5 mm.

(d-f) MKN45 cells depleted of MAPK4 were applied for MTT assay and colony formation. Quantification of cell colonies per well is shown. Scale bar, 10 mm.

(g-i) BGC-LM cells were infected with pLVX-MAPK4 lentivirus and subjected to MTT and colony formation analyses. Cell colonies per well were quantified. Scale bar, 5 mm.

KD, knockdown; OE, overexpression. Data are expressed as mean ± standard deviation. NS, not significant (Student's *t* test).

Supplementary Fig. 2

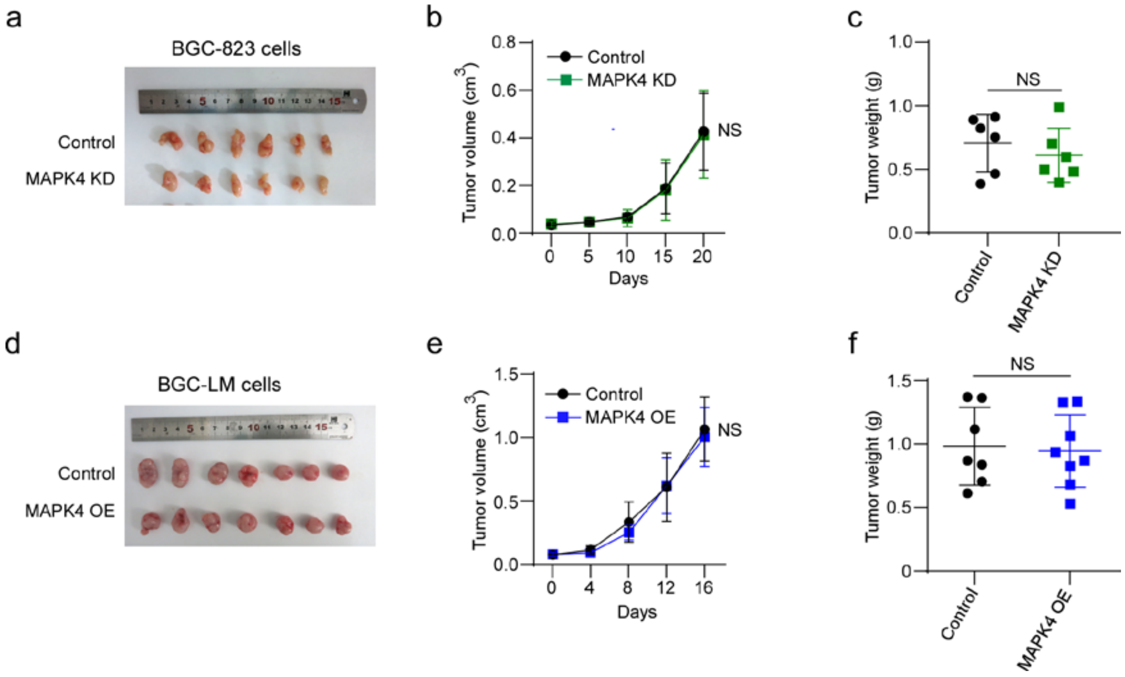

Supplementary Fig. 2. MAPK4 has no significant effect on the tumor growth of gastric cancer cells.

(a-c) BGC-823 cells depleted of MAPK4 were subcutaneously injected into SCID mice for tumor formation. Tumor volume was monitored at the indicated time points. Tumor weight was measured after dissection. Quantification of tumor volume and weight is shown.

(d-f) BGC-LM cells infected with pLVX-MAPK4 lentivirus or not were subcutaneously injected into SCID mice for tumor growth. Tumor volume was determined at the indicated time points. Tumor weight was recorded after dissection. Tumor volume and weight were quantified.

Data are expressed as mean ± standard deviation. NS, not significant (Student's *t* test).

Supplementary Fig. 3

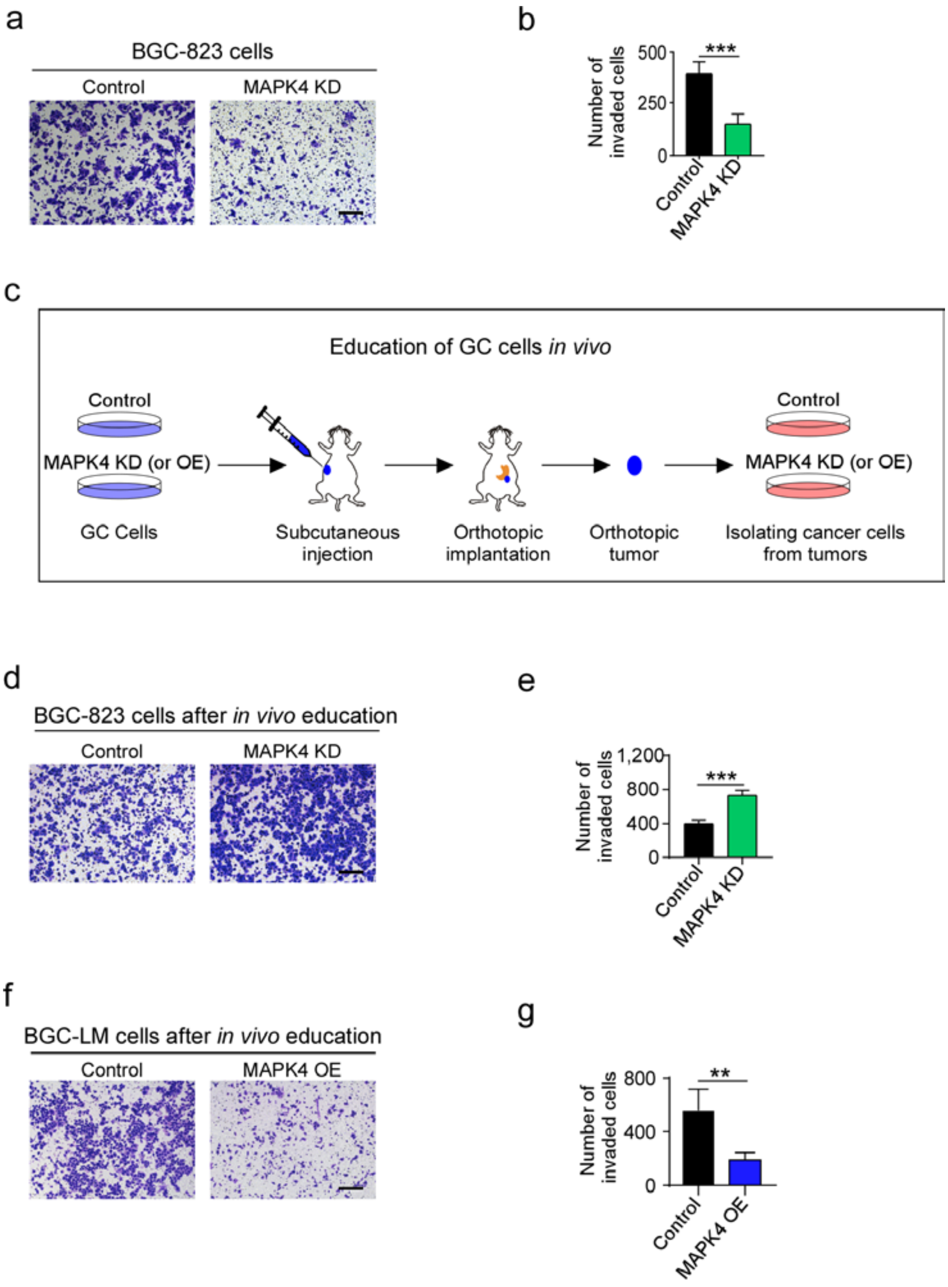

Supplementary Fig. 3. MAPK4 depletion suppresses gastric cancer cell invasion *in vitro* and promotes gastric cancer cell invasion *in vivo*.

(a, b) BGC-823 cells infected with the lentivirus targeting MAPK4 were subjected to Boyden chamber assays. Scale bar, 200 μm. Quantification of invaded cells per well is shown.

(c) Schematic illustration of the education process of lentivirus-infected gastric cancer cells in orthotopic implantation mouse model.

(d, e) Matrigel invasion analysis of control or MAPK4-depleted BGC-823 cells after education. Scale bar, 200 μm. Quantification of invaded cells per well is shown.

(f, g) Matrigel invasion analysis of control or MAPK4-overexpressing BGC-LM cells after education. Scale bar, 200 μm. Quantification of invaded cells per well is shown.

Data are expressed as mean ± standard deviation. ** $p < 0.01$; *** $p < 0.001$; NS, not significant (Student's t test).

Supplementary Fig. 4

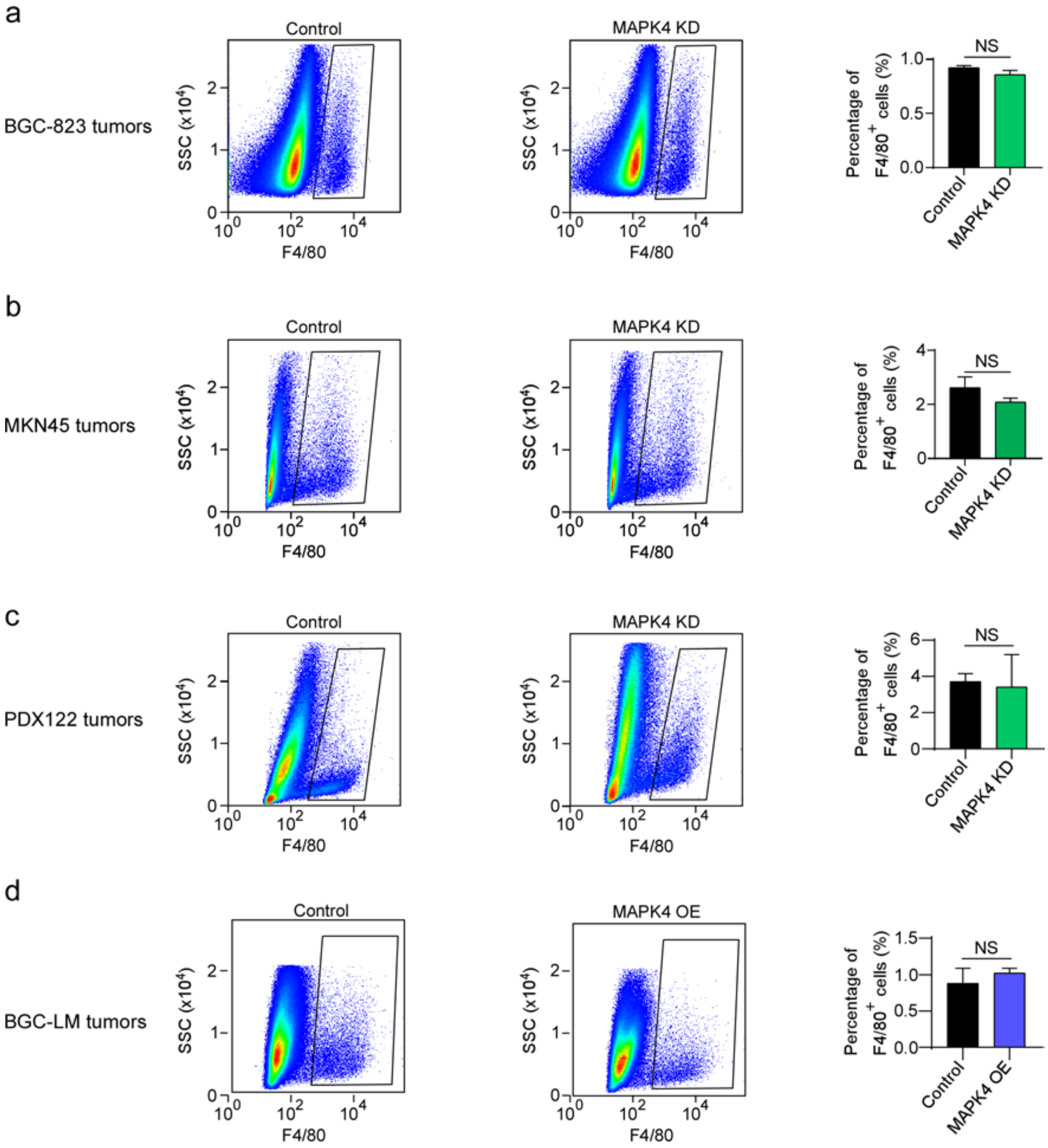

Supplementary Fig. 4. MAPK4 has no significant effect on macrophage infiltration in orthotopic gastric cancer tumors.

(a-c) Single-cell suspensions were prepared from orthotopic tumors of BGC-823, MKN45 and PDX122 cells depleted of MAPK4, and subjected to FCM analysis with anti-F4/80 antibody.

(d) Single-cell suspensions were isolated from orthotopic tumors of MAPK4-overexpressing BGC-LM cells and analyzed by FCM with anti-F4/80 antibody. Quantification of percentage of F4/80 positive cells is shown.

Data are expressed as mean ± standard deviation. NS, not significant (Student's *t* test).

Supplementary Fig. 5

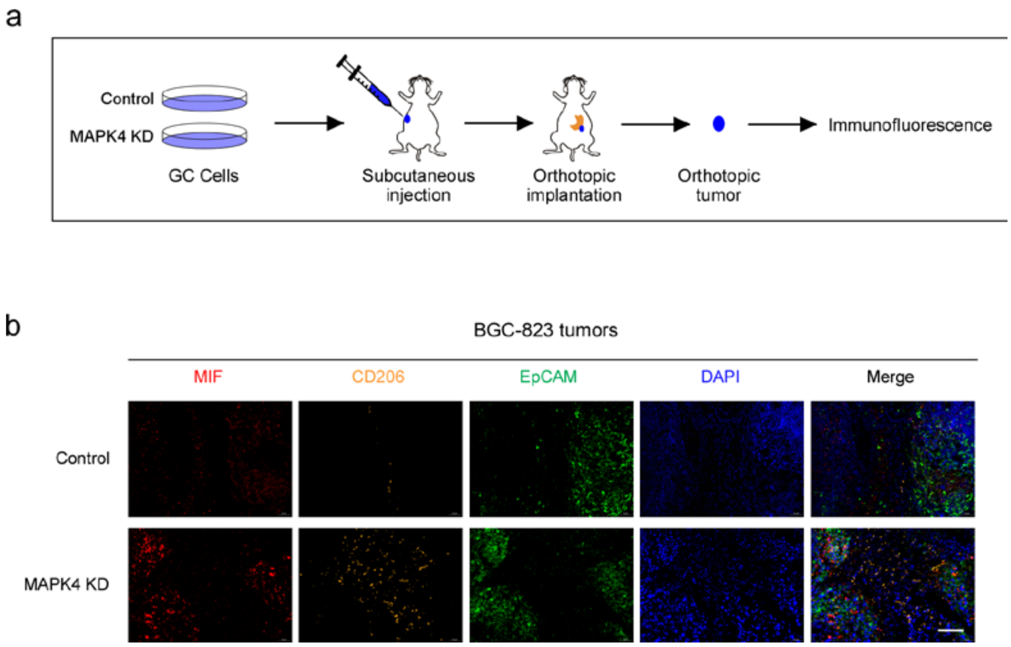

Supplementary Fig. 5. Depletion of MAPK4 increases the expression of MIF in cancer cells and the percentage of CD206-positive macrophages in orthotopic tumors.

(a) Schematic illustration of orthotopic implantation of control and MAPK4-depleted BGC-823 cells.

(b) Orthotopic tumors were analyzed by immunofluorescence with anti-MIF (red), CD206 (orange) and EpCAM (green) antibodies. Nuclei were stained with DAPI (blue). Scale bars, 100 μm.

Supplementary Fig. 6

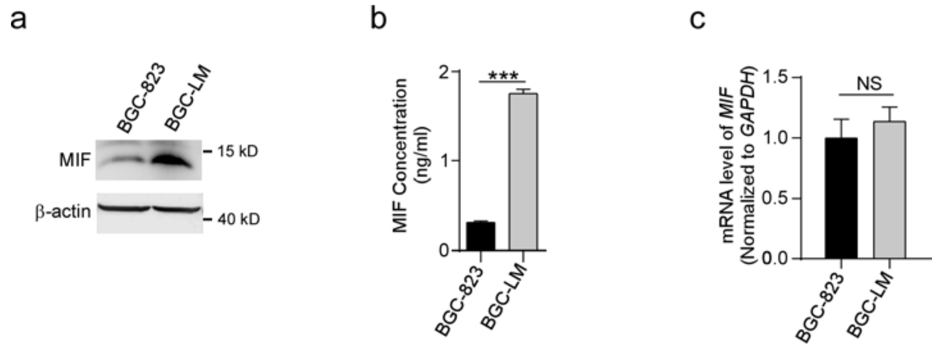

Supplementary Fig. 6. MIF protein level is reduced but mRNA level remains invariable in BGC-LM cells compared with BGC-823 cells.

(a) Western analysis of BGC-823 and BGC-LM cells with indicated antibodies.

(b) ELISA analysis of MIF levels in conditioned mediums from BGC-823 and BGC-LM cells.

(c) Quantitative RT-PCR analysis of *MIF* mRNA in BGC-823 and BGC-LM cells.

Data are expressed as mean ± standard deviation. *** $p < 0.001$; NS, not significant (Student's t test).

Supplementary Fig. 7

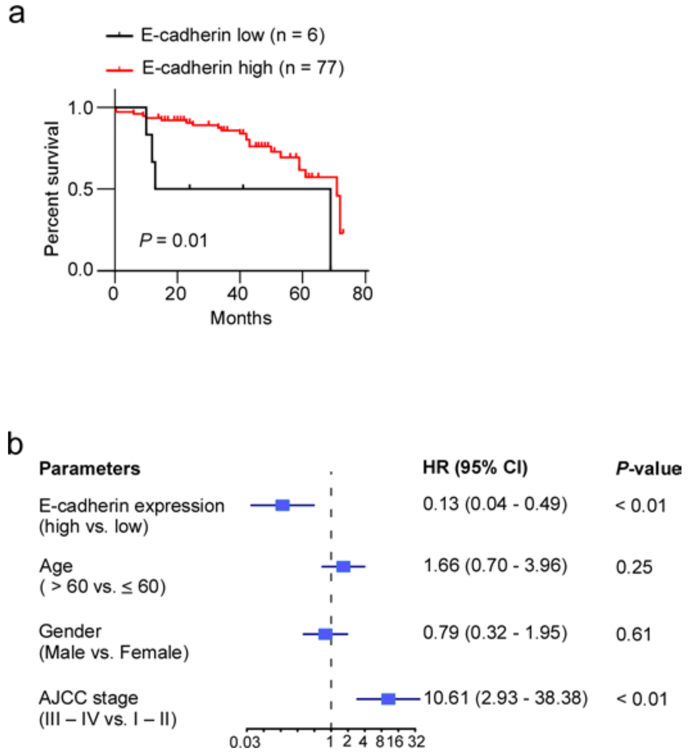

Supplementary Fig. 7. MAPK4 downregulation correlates with EMT in gastric cancer cells and tumor tissues from patients.

(a) Kaplan-Meier analysis of the correlation between E-cadherin expression in tumor tissues and overall survival of gastric cancer patients.

(b) Multivariable analysis of prognostic factors for gastric cancer patients.

Supplementary Table 1. Differentially secreted cytokines of BGC-823 cells depleted of MAPK4 by cytokine array analysis (Related to Fig. 5)

Cytokine	FC	Cytokine	FC	Cytokine	FC
SYN4	5.83	TSP2	0.67	S100A8	0.52
CXCL5	5.61	DR6	0.67	BDNF	0.49
PPBP	3.84	CXCL16	0.66	IL-6R	0.47
OSM	3.22	RBP4	0.66	IL-18	0.45
CD163	2.95	IL-1 F8	0.65	B2M	0.44
CXCL10	2.37	NSE	0.64	Tie-1	0.43
CCL5	1.98	PDGF-AB	0.63	Dtk	0.40
CD276	1.92	TIMP-1	0.61	CCL23	0.30
ADAM17	1.71	Fractalkine	0.60	bIG-H3	0.30
MIF	1.66	ErbB3	0.60	Albumin	0.26
TGFb2	1.63	JAM-B	0.60	Cathepsinb	0.18
Cystatin C	1.61	LAP	0.59	TIMP-4	0.14
Cystatin B	1.60	FSTL1	0.58		
FOLR1	1.59	FLRG	0.53		

NOTE: The fold change (FC) of each cytokine was calculated by comparing the average normalized fluorescence values of MAPK4-depleted group to that of control group. Fold changes greater than 1.5 or less than 0.67 are shown.

Supplemental Methods

Plasmids construction, transfection and lentivirus package

To generate overexpressing constructs of MAPK4 and MIF, full-length human MAPK4 and MIF cDNAs were amplified by PCR, and subcloned into the lentiviral vector pLVX-Puro with or without an N-terminal Flag, or Myc tag. The efficiency of overexpression was assessed by immunoblotting. The sequence of primers (5' to 3') are as follows: *MAPK4* forward primer, ATGGCTGAGAAGGGTGACTG; *MAPK4* reverse primer, TCACCACCTTTCTTTGGAGAAG; *MIF* forward primer, TGCCATCATGCCGATGTTC; *MIF* reverse primer, TTAGGCGAAGGTGGAGTTG.

To generate shRNA constructs of MAPK4 and MIF, oligos targeting mRNA of *MAPK4* or *MIF* were synthesized and cloned into the lentiviral vector pGLV3/H1/GFP. To generate cells stably expressing shRNAs, HEK293T cells were transfected with the indicated lentivirus expression vector and viral packaging constructs. Transient plasmid transfection was carried out with PolyJet (SignaGen Laboratories) DNA transfection reagent according to the manufacturer's protocol. The viral medium was collected, filtered and mixed with 1 μg/ml polybrene (Sigma) to infect the target cells. After 24h infection, cells were treated with 1 mg/mL of puromycin (Thermo Scientific) for 1 week. The efficiency of downregulation was assessed by immunoblotting. The shRNA sequence targeting *MAPK4* (5' to 3'): GCTTCGGTGTCAATGGTTTGG. The shRNA sequence targeting *MIF* (5' to 3'): GGACAGGGTCTACATCAACTA.

The siRNA transfection was performed using GenMute (SignaGen Laboratories).

The efficiency of transient knockdown was assessed by immunoblotting 48 hours after infection. The siRNA sequences are as follows:

Genes	siRNA sequences (5'-3')
Snail	GCCUUCAACUGCAAAUACU
Snail	CUUUGAGCUACAGGACAAA
Slug	GCUUCAAGGACACAUUAGA
Slug	CCUGCACAAACAUGAGGAA
Twist	GCAAGAUUCAGACCCUCAA
Twist	GGAGUCCGCAGUCUUACGA
Zeb1	GGCGGUAGAUGGUAAUGUA
Zeb1	GUCGCUACAAACAGUUGUA
Zeb2	GACCACUCCAGGAGUAAUA
Zeb2	CAGCCCUUUAGGAGUUCAU

Reverse transcription and qRT-PCR

Cell samples were lysed using TRIzol reagent (Invitrogen). Tissue samples were grounded to fine powder in liquid nitrogen and lysed using TRIzol reagent (Invitrogen). Total RNA was extracted according to the manufacturer's protocol. Reverse transcription of RNA to cDNA was performed by reverse transcription polymerase chain reaction kit (Vazyme). qRT-PCR using SYBR Green (Vazyme) was performed on a LightCycler 480 instrument (Roche). The mRNA levels of human genes or mouse genes were assessed by qRT-PCR. Each qRT-PCR reaction was independently repeated at least three times to ensure reproducibility. The data were analyzed by $2^{-\Delta\Delta CT}$ method [1]. The primers for qRT-PCR were designed by online tool (Sigma) as follows.

Genes	Species	Forward primer (5'-3')	Reverse primer (5'-3')
MAPK4	Human	CTGAAGTGAACAGTGAAG	GAGTATGGGCTCATGTAG
E-cadherin	Human	AGGCCAAGCAGCAGTACATT	ATTCACATCCAGCACATCCA

N-cadherin	Human	AGGTTTGCCAGTGTGACTCC	TGATGATGCAGAGCAGGATG
MIF	Human	GGACAGGGTCTACATCAACTA	TCTTAGGCGAAGGTGGAG
GAPDH	Human	GGAGCGAGATCCCTCCAAAAT	GGCTGTTGTCATACTTCTCATGG
Arg1	Mouse	AGAAGGTCTCTACATCACA	TACTCTTCACCTCCTCTG
Gapdh	Mouse	AGGTCGGTGTGAACGGATTTG	TGTAGACCATGTAGTTGAGGTCA

Western blotting and immunoprecipitation

For western blotting, cells were harvested in cold PBS and lysed in cold radioimmunoprecipitation assay (RIPA) buffer (50 mM Tris at pH 7.5, 1% Triton X-100, 0.5% deoxycholate, 150 mM NaCl, 10 mM EDTA) containing protein inhibitor cocktail (Roche). Proteins were separated by SDS-PAGE and transferred to PVDF membranes. Membranes were incubated with primary antibodies and HRP-conjugated secondary antibody. The following antibodies were used: anti-human MAPK4 (1:500, 26102-1-AP, Proteintech), anti-human E-cadherin (1:1000, 3195, CST), anti-human MIF (1:500, 20415-1-AP, Proteintech), anti-human Snail (1:1000, 13099-1-AP, Proteintech), anti-human Slug (1:1000, 12129-1-AP, Proteintech), anti-human Twist (1:500, 25465-1-AP, Proteintech), anti-human Zeb1 (1:500, 21544-1-AP, Proteintech), anti-human Zeb2 (1:500, 14026-1-AP, Proteintech), anti-human N-cadherin (1:500, 22018-1-AP, Proteintech), anti-human β-actin (1:1000, A5441, Sigma) and HRP-conjugated secondary antibody (1:5000, Thermo Scientific). The protein signals on the membranes were detected with ECL (Thermo Scientific). For immunoprecipitation, cells were lysed in cold lysis buffer (20 mM Tris at pH 8.0,

150 mM NaCl, 0.5% NP-40, 5 mM EGTA, 1.5 mM EDTA, 0.5 mM Na3VO4). Cell lysates were incubated with the anti-Flag or anti-Myc antibodies (CST) and protein A/G-agarose beads (Santa Cruz). The immunoprecipitates were subsequently analyzed by western blotting using the indicated antibodies.

Cell proliferation

For MTT assay, cancer cells were seeded in 96-well plates for the indicated times (0, 24, 48, 72, or 96 hours), and relative cell number was measured by 3-(4,5-Dimethylthiazol-2-yl)-2,5-diphenyltetrazolium bromide (Sigma) as described previously [2]. For colony formation experiment, cancer cells were seeded in 12-wells plates at an appropriate density, and incubated for one to two weeks until the colonies were visible to the naked eye. The colonies were stained with crystal violet (Sigma) and quantified.

Cell education *in vivo*

For education of gastric cancer cells, gastric cancer cells were infected with indicated lentivirus, subcutaneously injected, and orthotopically implanted into mice. After 3-4 weeks, orthotopic tumors were digested into single-cell suspensions and cancer cells were isolated and purified from the suspensions.

Isolation and stimulation of BMDMs from mice

L929 cells (kind gift from Dr. Qingqing Wang, Zhejiang University) were cultured in

DMEM medium (Corning) supplemented with 10% FBS (Hyclone) and penicillin/streptomycin. Conditioned medium was collected and filtered to make BMDMs growth medium.

BMDMs were isolated and cultured as described previously [3]. Briefly, femur and tibia bones were separated from 6-week-old SCID mice. The bone marrow cells were isolated and cultured in BMDMs growth medium (IMDM medium with 10% FBS and 15% conditioned medium of L-929 cells) for 7 days. BMDM maturation was measured by detecting the expression of F4/80 with flow cytometry.

Immunohistochemistry and immunofluorescence

Orthotopic tumor tissues or liver tissues were fixed in 4% buffered formalin immediately after mice dissection. The fixed tissues were then dehydrated in ethanol, embedded with paraffin and sectioned at 6 μm. For H&E staining of liver tissues, sections were incubated with hematoxylin solution (Sigma) and eosin solution (Sigma) as described previously [4]. For immunohistochemistry or immunofluorescence of tumor tissues, sections were blocked by 5% goat serum (Thermo Scientific) in PBS. Then the sections were incubated with primary antibodies and HRP- conjugated antibodies or fluorescent-conjugated antibodies. The following antibodies were used: anti-human MAPK4 (1:100, 26102-1-AP, Proteintech), anti-human E-cadherin (1:1000, 3195, CST), anti-mouse F4/80 (1:100, 14-4801, eBioscience), anti-mouse CD206 (1:100, MCA2235T, Bio-rad), HRP-conjugated secondary antibody (1:200, SA00001-2, Proteintech), Alexa Fluor 488 conjugated secondary antibody (1:200,

A-11008, Invitrogen) and Alexa Fluor 555 conjugated secondary antibody (1:200, A-21431, Invitrogen). Hematoxylin (Sigma) or DAPI (Invitrogen) were used to stain cell nucleus. Images were collected from a confocal microscope (LSM 880, Zeiss) and processed with ZEN image software. Tissue microarrays were scanned with a digital slide scanner (Pannoramic MIDI, 3D HISTECH) after staining, and then processed to the Pannoramic viewer software. Intensity of staining in cells was automatically calculated by Quant center software. H-score was acquired according to the formula: H-score = (percentage of cells of weak intensity ×1) + (percentage of cells of moderate intensity ×2) + (percentage of cells of strong intensity ×3).

References

1. Livak, K. J. & Schmittgen, T. D. Analysis of relative gene expression data using real-time quantitative PCR and the 2(-Delta Delta C(T)) Method. *Methods* **25**, 402-408 (2001).
2. Zhuo, W. *et al.* Long Noncoding RNA GMAN, Up-regulated in Gastric Cancer Tissues, Is Associated With Metastasis in Patients and Promotes Translation of Ephrin A1 by Competitively Binding GMAN-AS. *Gastroenterology* **156**, 676-691 e611 (2019).
3. Weischenfeldt, J. & Porse, B. Bone Marrow-Derived Macrophages (BMM): Isolation and Applications. *CSH Protoc*. **2008**, pdb prot5080 (2008).
4. Fischer, A. H., Jacobson, K. A., Rose, J. & Zeller, R. Hematoxylin and eosin staining of tissue and cell sections. *CSH. Protoc* **2008**, pdb prot4986 (2008).
